# Household food insecurity, diet, and weight status in a disadvantaged district of Ho Chi Minh City, Vietnam: a cross-sectional study

**DOI:** 10.1186/s12889-015-1566-z

**Published:** 2015-03-08

**Authors:** Thuy Ngoc Vuong, Danielle Gallegos, Rebecca Ramsey

**Affiliations:** Department of Epidemiology, the Institute of Hygiene and Public Health, 159, Hung Phu Street, Ward 8, District 8, Ho Chi Minh City, Vietnam; School of Exercise and Nutrition Sciences, Queensland University of Technology, Victoria Park Avenue, Kelvin Grove, Queensland, 4059 Australia

**Keywords:** Household food insecurity, Diet, Weight, Health, Vietnam

## Abstract

**Background:**

Food security exists when all people, at all times, have physical, economic and socially acceptable access to safe, sufficient, and adequately nutritious food in order to meet their dietary needs for an active and healthy life. For high income countries and those experiencing the nutrition transition, food security is not only about the quantity of available food but also the nutritional quality as related to over- and under-nutrition. Vietnam is currently undergoing this nutrition transition, and as a result the relationship between food insecurity, socio-demographic factors and weight status is complex. The primary objective of this study was to therefore measure the prevalence of household food insecurity in a disadvantaged urban district in Ho Chi Minh City (HCMC) in Vietnam using a more comprehensive tool. This study also aims to examine the relationships between food insecurity and socio-demographic factors, weight status, and food intakes.

**Methods:**

A cross-sectional study was conducted using multi-stage sampling. Adults who were mainly responsible for cooking were interviewed in 250 households. Data was collected on socioeconomic and demographic factors using previously validated tools. Food security was assessed using the Latin American and Caribbean Household Food Security Scale (ELCSA) tool and households were categorized as food secure or mildly, moderately or severely food insecure. Questions regarding food intake were based on routinely used and validated questions in HCMC, weight status was self-reported.

**Results:**

Cronbach’s alpha coefficient was 0.87, showing the ELCSA had a good internal reliability. Approximately 34.4% of households were food insecure. Food insecurity was inversely related to total household income (OR = 0.09, 95% CI = 0.04 - 0.22) and fruit intakes (OR = 2.2, 95% CI 1.31 - 4.22). There was no association between weight and food security status.

**Conclusions:**

Despite rapid industrialization and modernization, food insecurity remains an important public health issue in large urban areas of HCMC, suggesting that strategies to address food insecurity should be implemented in urban settings, and not just rural locations. Fruit consumption among food insecure households may be compromised because of financial difficulties, which may lead to poorer health outcomes particularly related to non-communicable disease prevention and management.

## Background

Food and nutrition security exists *“*when all people at all times have physical, social and economic access to food, which is safe and consumed in sufficient quantity and quality to meet their dietary needs and food preferences, and is supported by an environment of adequate sanitation, health services and care, allowing for a healthy and active life*”* [1]. Conversely, food insecurity exists when access to or availability of such foods is limited, or when these foods cannot be accessed by socially acceptable means. Of note, is that food security does not only relate to the food quantity but also quality, with the emphasis on foods that promote and maintain health and wellbeing.

Food insecurity may have adverse impacts on the nutritional and health status of individuals [2,3] and these impacts may vary between high income and low to middle income countries. Among children in high income countries, food insecurity may result in deficits in physical and psychological development and lower academic achievement [4,5]. Among adults, food insecurity may be associated with poor dietary choices, and consequently overweight and obesity (primarily among women), poorer health, depression, and an increased risk of diet-related chronic conditions, such as cardiovascular diseases and diabetes [2,6]. In low to middle income countries, food insecurity among adults and children has been associated with poor dietary intakes, malnutrition, and underweight (as opposed to obesity as seen in high income countries). However, as several low to middle income countries begin to undergo the nutrition transition, diet and health factors associated with food insecurity may begin to mimic those of high income countries, resulting in a ‘triple burden of disease’ in which these countries experience high rates of obesity, chronic disease and mental health issues as well as malnutrition and communicable diseases [7]. Despite this, there exist a limited number of studies investigating the associations between food security, diet and weight status in urban areas, in countries experiencing the nutrition transition [2,7].

Currently, investigations into household food insecurity in Vietnam are limited, relying on indirect indicators such as the poverty rate or Hunger Index [8], in lieu of more direct, comprehensive tools [2,9]. Previous studies have suggested a dissonance between the prevalence of food insecurity estimated by direct methods compared to indirect methods [9,10]. Based on poverty rates, it is estimated that the prevalence of food insecurity among the population of Vietnam in 2009 was around 14% with no reporting of differences between urban and rural areas [11]. In 2002, the rural food poverty rate was estimated at 13.6% compared to 1.9% within urban areas [12]. However, the use of rates of undernourishment as a proxy for food insecurity revealed a prevalence estimate 7% higher compared to the poverty rate [9]. This poses a problem as targeted interventions aimed at those defined conventionally as “poor”, could potentially exclude large numbers of food insecure households. It should also be noted that undernourishment only deals with average caloric intake, while food security requires both adequate quantity and quality of foods. In addition, given the paradoxical relationship between food insecurity and overweight/obesity in high income countries which is also likely to be evident in countries undergoing the nutrition transition, the measurement of undernourishment may not accurately reflect food insecurity rates. Thus, there is a need for a more specific assessment of food insecurity in order to assist policy-makers establish equitable and effective public programs.

With economic growth, Vietnam may be able to provide enough food for its population [13]. Given this potential, it could be assumed that food insecurity is no longer an issue, especially in economically developed areas. However, this may not be the case as sufficient amounts of food at the population level do not necessarily guarantee equitable distribution at the household level, both between and within households. Specifically, disadvantaged urban populations may be at risk of food insecurity due to their lack of access to agricultural resources compared to their rural counterparts [14]. Additionally, recent financial crises leading to higher food prices and unemployment in large cities may also increase rates of food insecurity among disadvantaged urban households [14].

Despite the high likelihood of food insecurity among the disadvantaged urban population [13,15], the government has made food security a major agricultural policy aim [16]. As such, most food security policies and programs rightly heavily invest in agriculture to ensure the supply of food for the whole population [13]. These programs frequently prioritize ethnic minority groups (those living in the mountains of the north and in the central highlands who include Khmer Krom, Hoa, Lao, the Mon-Khmer, the Tay) and the disadvantaged in remote and rural areas, because there is an assumption that food insecurity is more prevalent in these areas due to a lack of resources to obtain sufficient quantities of food [8,17]. These programs have effectively increased the amount of food available and as a result improved food access. While it is essential that these policy initiatives need to be maintained at the national level, they may however, be insufficient to ensure equitable access, availability, and affordability for urban poor households.

This research aimed to identify the prevalence of food insecurity among an urban, disadvantaged population in HCMC, using a more comprehensive and direct measure, the Latin American and Caribbean Household Food Security Scale (ELCSA) tool [18]. This tool has been adapted from the USDA Food Security Survey Module (FSSM) [19] and has been applied in countries with similar development indexes to Vietnam [20-22]. In addition, the potential associations between food insecurity and socio-demographic attributes, dietary factors and weight status were also investigated.

## Methods

### Ethical approval

This study was approved by the Queensland University of Technology Human Research Ethics Committee (UHREC), Australia (approval number 1100001491) and the Institute of Hygiene and Public Health (IHPH) of HCMC, Vietnam.

### Research setting

HCMC is located in the South of Vietnam. It is the largest city with an estimated population of eight million people [23]. District eight (D8) is one of the poorest districts in HCMC with a large number of people living in slums and shanties [24]. In 2011, approximately 5,500 households with 29,504 people in this district lived below the national poverty line [25].

### Sample and sampling methodology

Sample size calculations yielded a required sample size of 250 individuals to determine statistically significant differences in rates of food insecurity with 90% power. This assumed a non-response rate of 10% [26].

Two-hundred and fifty adults who were responsible for cooking in their households in D8 were identified and approached using a multi-stage sampling method. The district is divided into 16 wards. Three out of the 16 wards, were randomly selected (Wards 2, 8, and 15) and households were subsequently randomly sampled. The total number of households in each ward was selected by Probability Proportional to Size sampling with 100 households sampled from Wards 2 and 15; and 50 households sampled from Ward 8. The adult responsible for cooking and, where relevant, the youngest child was then selected from each household. A small gift valued at $1AUD was given to participants to thank them for participation.

### Data collection

One week in advance of data collection a one day training session in interview techniques and use of the questionnaire was undertaken for 12 students majoring in public health at the Ho Chi Minh City University of Medicine and Pharmacy. These data collectors went door-to-door, in pairs, to undertake the interviews. The interviews were mostly conducted in the evenings after dinner and on the weekends to ensure the majority of participants were present in the household.

#### Socio-economic and demographic characteristics

Participants’ age, gender, ethnicity, level of education, place of birth, household income, and family type were recorded. A household was defined as “people who sleep under the same roof and share food with each other” [27]. To determine place of birth, participants were asked to record the province in which they were born. These were then categorised into “HCMC” and “other”. Age of the participant was reported as a continuous variable, then categorised into 18–44 years, 45–54 years, 55–64 years or 65 or older years for analyses. Ethnicity was recorded as either Kinh (the majority group) or “other”. Total household income was categorized into “ ≤ the poverty line (PVL)” versus “ > PVL” according to the PVL for urban areas set by the Ministry of Labour Invalids and Social Affairs (MOLISA) [28]. Urban households who are considered poor have an income of one million VD (AUD 45) or less per month per household [28].

Additionally, ownership of 12 high-value items derived from the Bureau Statistics were used to evaluate household income level [29]. This is an indirect way of assessing household income, however is considered a practical means compared to direct questioning. Each affirmative response to the aforementioned items in the households received a score of one. Values were then summed and households were ranked accordingly as low income (the total score was less than 4), medium income (a score of 4–8), and high (a score of 9–12).

#### Food security status

Food security status was assessed using the 15-item Latin American and Caribbean Household Food Security Scale (ELCSA), which is a modified version of the USDA FSSM [21]. Households are classified as food insecure if they respond affirmatively to any questions [30]. Households are further categorized as mildly, moderately, and severely food insecure if they have 1–5, 6–10, and 11+ affirmative responses respectively [30].

The English version of the ELCSA was translated into the Vietnamese language and pretested among 30 local people to assess face and content validity, and where required make minor adjustments to wording to improve comprehension before data collection. Although this tool has not yet been formally validated amongst a Vietnamese population, it yielded high internal and external reliability in Mexico, Colombia, Ecuador, Guatemala, El Salvador, Honduras, and Nicaragua [20]. Similar to Vietnam, most of these countries are undergoing the nutrition transition [31] and have similar development indexes [32]. This tool had a reliability of Cronbachs alpha = 0.87 within the current sample, indicating high internal reliability.

#### Weight status

Weight status was assessed by calculating body mass index (BMI). Participants self-reported their weight and height, which were used to calculate BMI as per the standard equation (weight(kg)/height(m)^2^). The Asian specific BMI cutoff of 23.0 kg/m^2^ was used to define overweight and obesity among adults [33]. Parental proxy-report was also used to obtain weights and heights for children. BMI was then calculated as per standard protocol and assessed against World Health Organisation child-specific BMI cut-off values to classify children as either underweight/healthy weight or overweight/obese [34].

#### Vegetable and fruit intakes

Fruit and vegetable intakes were assessed using short-answer questions that asked “How often do you eat vegetable/fruit *per week*?” [2] Responses to this item were categorized into “everyday” and “< everyday” for the purpose of data analyses. These short-answer intake questions have previously been used in a study to validate food frequency questionnaires among Vietnamese in HCMC [35].

#### Meat consumption

Participants were asked to report on their usual weekly consumption of processed meats, red meat (pork and beef), fish and other seafood, chicken and other poultry, for example “How many times per week do you eat red meat?” [2] Responses were coded as “Yes” (1–2 times/week + ≥ 3 times/week) and “No” (never/rarely”) for analyses.

#### Fast foods and take-away foods

Take away and fast food consumption was assessed using items measuring the frequency of consumption of sweet sticky rice, fried chicken and potato chips, hamburgers, pizza, and cakes. These foods were the most popular takeaway items consumed in HCMC [35]. Responses were coded as “Yes” (1–2 times/week + ≥ 3 times/week) and “No” (never/rarely”).

#### Soft drink and alcohol

Drinks included items measuring the frequency of consumption of carbonated soft drinks and alcohol asked as separate questions. Responses were coded as “Yes” (1–2 times/week + ≥ 3 times/week) and “No” (never/rarely”).

### Data analyses

Data were entered into Epi Data 3.0 and analysed using Stata 11.0. Chi-square analyses were used to investigate bivariate associations between food insecurity and the socio-demographic, dietary and weight variables. Binary logistic and multiple linear regressions were then applied to investigate associations between food insecurity and diet, and weight factors, that were identified as being significant at the bivariate level, controlling for socio-demographic characteristics.

## Results

### Socio-demographic characteristics

The demographic characteristics of the sample and the total population of HCMC are summarised in Table 1. The majority of respondents were female, of Kinh (the majority group) ethnicity, had completed secondary school, were married and from households in the medium tertile for income. As the respondents were providing answers at the household level, the higher proportion of women respondents was unlikely to affect the representativeness of the sample.

**Table 1:** **Socio-demographic characteristics of the study sample from District Eight (n = 250) compared to the total population of the HCMC**

**Characteristics**	**Total study sample (%)**	**Total population of HCMC (%)** ^*^
**Age**		
18-44	45.0	54.0
45-64	45.0	17.0
≥65	10.0	5.0
**Gender**		
Female	89.0	52.0
Male	11.0	48.0
**Ethnicity**		
Kinh	95.0	93.0
Others (Chinese, the Tay, Cham, ect.)	5.0	7.0
**Education**		*% (Urban population)* ^†^
Never went to school	5.2	3.1
Completed primary school (5 years of schooling)	28.0	25.9
Completed secondary school (9 years of schooling)	41.2	46.6
Completed high school (12 years of schooling)	19.6	n/a
Completed college or above (>12 years of schooling)	6.0	15.2
**Place of birth**		
HCMC	57.2	n/a
Others	42.8	
**Settlement time**		
≤10 years	26.0	n/a
>10 years	74.0	

^*^ The 2010 Viet Nam Population and Housing Census and 2010 Statistical Yearbook of Ho Chi Minh City.

^†^Data for “Education” were unavailable for HCMC, but available for urban areas.

n/a: not available.

### Food insecurity

Over one-third (34.4%) of households reported some degree of food insecurity. Mildly food insecure households accounted for the highest percentage (21.6%) with 12.0% identifying as moderately food insecure. There was an extremely low proportion (0.8%) of severely food insecure households.

Table 2 summarises the associations between food insecurity and socio-demographic characteristics of the households. At the bivariate level, food insecurity was significantly associated with age (p = 0.01), education (p < 0.01), settlement time (p = 0.02), and total household income (p < 0.01) using Chi-square test. After adjustment for appropriate covariates, the only significant association that remained was between food insecurity and total household income. Medium and high income households were 60% and 93% less likely to experience food insecurity respectively, compared to their lower income counterparts (p < 0.01).

**Table 2:** **Food insecurity by socio-demographic characteristics among households in District Eight in HCMC (n = 250)**

**Characteristics**	**Food insecurity status**			
	**Food insecure (%)**	**Food secure (%)**	P value**	**OR (95% CI)**
**Age** ^**†**^^**†**^			0.01*	
18-44	32.5	51.2		1.00
45-64	52.3	41.4		1.63 (0.68-3.94)
≥65	15.1	7.3		1.71 (0.91-4.02)
**Gender**			0.18	
Male	23.0	77.0		
Female	36.2	63.8		
**Education** ^**†**^			0.00*	
Never went to school	4.2	6.8		1.38 (1.09-1.83)
Completed primary school	38.6	21.9		1.41 (1.22-1.63)
Completed secondary school	44.3	39.0		1.27 (1.12-1.44)
Completed high school or above	10.2	34.7		1.00
**Ethnicity**			0.83	
Kinh	34.0	66.0		
Others	40.0	60.0		
**Settlement time** ^**‡**^			0.02*	
≤10 years	82.6	69.5		2.13 (1.08-4.31)
>10 years	30.5	17.4		1.00
**Employment**			0.25	
**Family size**			0.95	
**Living arrangement**			0.19	
**Total income by urban poverty lines**			0.18	
**Total household income by house items** ^**§**^			0.00*	
Low	100.0	0.0		7.28 (2.99-17.69)
Medium	44.0	60.5		3.14 (1.24-4.33)
High	6.7	93.3		1.00

*Significant at p < 0.05.

**P-value for chi-square (difference between food secure and food insecure households).

^**†**^^**†**^Adjusted for education.

^†^Adjusted for age and total income.

^‡^Adjusted for age.

^§^Adjusted for age and education.

Table 3 summarises the bivariate and multivariate associations between food insecurity and dietary intakes. At the bivariate level, food insecurity was significantly associated with lower intakes of fruit (p = 0.02), processed meat (p = 0.04), fresh chicken and other poultry (p < 0.01). After adjustment for potential confounders, only intakes of fruit remained associated with food insecurity (OR and 95% CI = 2.2 (1.31 - 4.22).

**Table 3:** **Associations between food insecurity and dietary intakes among households in District Eight, HCMC (n = 250)**

		**Food security status**			
**Dietary intakes**	**Total sample (%)**	**Food insecure** **(%)**	**Food secure** **(%)**	p-value**	**OR (95% CI)**
**Fruit intake**				0.02^*^	
Less than everyday	49.2	36.0	9.1		2.2 (1.31 - 4.22)
Everyday	50.8	64.0	90.9		1.00
**Vegetable**				0.17	
Less than everyday	14.4	18.6	12.2		
Everyday	85.6	81.4	87.8		
**Processed meat**				0.04*	
Yes	18.4	11.6	21.9		1.00
No	81.6	88.4	78.1		1.88 (0.87 - 4.03)
**Red meat (pork, beef)**				0.91	
Yes	86.4	86.1	86.6		
No	13.6	14.0	13.4		
**Fresh chicken and other poultry**				0.00^*^	
Yes	61.2	50.0	67.1		1.00
No	38.8	50.0	33.0		1.18 (0.65 -2.15)
**Fish and other seafood**				0.19	
Yes	81.6	86.1	79.3		
No	18.4	14.0	20.7		
**Sweet sticky rice**				0.76	
Yes	26.8	27.9	26.2		
No	73.2	72.1	73.8		
**Cakes**				0.88	
Yes	20.4	79.1	79.9		
No	79.6	20.9	20.1		
**Fried chicken and potato chips**				0.10	
Yes	7.2	3.5	9.1		
No	92.8	96.5	90.8		
**Hamburger**				0.77	
Yes	5.2	4.7	5.5		
No	94.8	95.4	94.5		
**Pizzas**				0.75	
Yes	5.2	5.8	4.9		
No	94.8	94.2	95.1		
**Soft drinks**				0.50	
Yes	34.4	37.2	32.9		
No	65.6	62.8	67.1		
**Beer**				0.21	
Yes	23.2	18.6	25.6		
No	76.8	81.4	74.4		
**Alcohol**				0.39	
Yes	10.4	8.1	11.6		
No	89.6	91.9	88.4		

^*^Significant at p < 0.05.

**P-value for chi-square (difference between food secure and food insecure households).

Weight status and its associations with food security status are summarised in Table 4. Overall, the prevalence of underweight among adults was low at only 8%. Individuals from food insecure households reported higher rates of underweight compared to their food secure counterparts (13.6% versus 8.3% among adults and 43.5% versus 39.6% among children). The prevalence of overweight and obesity among adults was high (approximately 33% and 10% respectively). Among children, the prevalence of overweight and obesity was low (less than 5% among boys and girls). Weight status was not significantly associated with food insecurity among adults or children (p > 0.05).

**Table 4:** **The associations between food insecurity and weight status among households in District Eight, HCMC (n = 250)**

		**Food security status**		
	**Total sample (%)**	**Food insecure (%)**	**Food secure (%)**	p-value**
**BMI Adults**				0.40
Underweight	8.4	13.6	8.3	
Healthy weight	49.2	56.8	52.3	
Overweight/obesity	42.4	29.5	39.4	
**BMI Children**				
**Male children**				0.90
Thinness^§^	2.6	43.5	39.6	
Healthy weight	93.4	43.5	49.1	
Overweight/obese^¶^	4.0	13.0	11.3	
**Female children**				0.79
Thinness^§^	4.1	61.9	53.9	
Healthy weight	91.8	38.1	42.3	
Overweight/obese^¶^	4.1	0.0	3.9	

^§,¶,^International BMI cutoff points to define thinness, overweight and obesity in children and adolescents were applied [36].

Statistical significance: p < 0.05.

**P-value for chi-square (difference between food secure and food insecure households).

## Discussion

Findings of the current study suggest that food insecurity is a salient issue among this disadvantaged urban population of HCMC, identifying a prevalence rate of 34% which is markedly higher than the 14% among the entire Vietnamese population (recorded in the latest national report in 2010), and the 1.9% identified in urban areas in 2002 [37]. This finding supports previous evidence, suggesting that rates of food insecurity are greater among disadvantaged communities than the general population [2,37-39]. It is also consistent with results from countries undergoing the nutrition transition, namely India [40], the Philippines [41], and Guatemala [42], which have suggested that disadvantaged urban populations, and those populations in marginal rural areas, are at risk of food insecurity in countries undergoing the nutrition transition. This finding suggests that the true burden of food insecurity among poor households living in densely populated areas may be as serious as those having inadequate resources to access foods in rural locations and among ethnic minority groups.

Consistent with previous studies, we identified a significant, inverse association between food insecurity and fruit intakes. This association may be explained by changes in food prices. The price of food has increased significantly since the 2008 global food crisis [43], in particular fruit prices have risen sharply between 2006 and 2011 in HCMC. Urban households who are economically vulnerable are more affected by soaring food prices [44]. It is possible that due to financial constraints, disadvantaged urban households may opt for energy-dense foods, which are perceived to be more satisfying and better value for money, compared to their low energy counterparts, such as fruits and vegetables. The Vietnamese traditional diet is mostly rice, fish, and vegetables, plus pork or chicken if affordable [45]. Fruits may be perceived as having low energy density in relation to price, and as such considered a luxury among low income households [46,47]. This may raise a public health concern because limited consumption of fruits may result in lower intakes of some micronutrients and is implicated in the development of many non-communicable diseases [48,49].

There were no associations between food insecurity and intakes of meats, take away or fast foods, soft drinks, and alcohol at either the bivariate or multivariate level in our sample. Lower income urban populations are relatively ‘conservative’ with respect to the consumption of Western-style foods and potentially more “faithful” to traditional foods, contributing to very low consumption proportions of the Western-style foods [50]. In addition, these foods, for poor households in Vietnam, are considered luxury items [50]. This is consistent with tenets of the nutrition transition where these foods are more likely to be consumed by those with higher incomes in these countries [51,52].

A lack of association between food insecurity and consumption of take away or fast foods and soft drinks is consistent with the early stage of the nutrition transition when the costs of these foods are still high, and as such, less affordable for the lower income households. Furthermore, at the time of the data collection, there was still little penetration of Western-style foods into the Vietnamese market [53]. However, it should be noted that as the nutrition transition continues, these foods will be much more prevalent in the domestic market, and therefore the price of these foods will go down and they will become more affordable for disadvantaged households. Together with other factors, such as physical inactivity, this is likely to exacerbate obesity rates. Thus, we suggest that nutrition surveys should assess the consumption of take away or fast foods and sugar sweetened beverages regularly in order to establish appropriate plans and strategies to prevent obesity, in a timely fashion.

Interestingly, the present study found not only a high prevalence of food insecurity (34%), but also a high proportion of overweight/obesity among adults (42%). Although a statistically significant association between food insecurity and overweight/ obesity was not evident, trends existed whereby a higher proportion of those from food insecure households were overweight or obese compared to their food secure counterparts. This supports previous evidence which suggests that food insecurity and obesity coexist in the disadvantaged households in urban areas in developing countries [14,54-56]. In fact, as the Vietnamese economy continues to be integrated with international trading markets, the costs of processed foods will become less expensive. Cheaper prices may lead to increased consumption of these foods which when accompanied with lower levels of physical activity may contribute to the food insecurity – obesity paradox in urbanized areas. Thus, we suggest further research to explore this complex issue.

Our findings also suggest a higher rate of underweight among adults from food insecure households (compared to those from food secure households), however these results did not reach statistical significance. Given that self-reported weight and height tends to underestimate weight status the relationship between weight and food security in urban areas, in countries undergoing the nutrition transition warrants further investigation [57]. Food insecurity may still predict underweight among socio-economically disadvantaged populations undergoing the nutrition transition [58]. The traditional energy deficits associated with food insecurity in countries experiencing the nutrition transition is likely to change. Households will potentially move away from small amounts of traditional foods, and corresponding underweight/malnutrition, to a situation in which food insecurity is associated with a surfeit of high fat, high sugar foods and overweight/malnutrition. Regardless, both of these forms of food insecurity are characterized by a household’s reduced ability to access affordable foods needed for health.

Consistent with previous studies, we did not identify a significant association between food insecurity and child weight status. In addition, the estimates of thinness, overweight, and obesity among children in previous studies [29,58] were higher than our findings. Research among low-income mothers in Canada has revealed that parents often ‘protect’ their children from food insecurity by compromising their own intakes first [59]. As such, it is possible that until more severe forms of food insecurity are reached, children will not experience any changes to their food intake, and consequently no changes to their weight status. Alternatively, our null finding could be due to a sole focus on the poor urban population, rather than a more socio-economically diverse sample, resulting in a homogenous sample in regards to weight status. Furthermore, it is possible that overweight and obesity among children may be underestimated as our study relied on parental proxy report of weight and height for children. Previous studies have suggested that the use of such self-reported data often results in an underestimation of weight and overestimation of height, resulting overall in an underestimation of BMI among children and subsequently an underestimation of the association between overweight and obesity and food insecurity [60-62]. If feasible, future investigations should use more objective methods to measure the weights and heights of children more accurately.

The results of the current study should be interpreted within the context of several limitations. The cross-sectional design makes it impossible to investigate the temporality of the associations identified and as such a causal relationship cannot be established. We relied on self-reported data and therefore response bias is a potential issue. Previous studies have suggested that the use of such self-reported data often results in an underestimation of weight and overestimation of height, resulting overall in an underestimation of BMI among both adults and children [60-62]. In particular, this may have resulted in an underestimation of the true association between food insecurity and overweight and obesity in the current sample. Where possible, studies should seek to collect objective measurements of these variables to more accurately determine whether associations between food security and weight status exist.

Finally, information regarding the specific types of public assistance program participation was not collected. As a result, the survey could have overestimated food insecurity if the respondents had expectations that the answers would influence their access to government support.

## Conclusions

This research is the first to assess household food insecurity using the ELCSA in Vietnam. Although poverty may be deeper and more widespread in rural and ethnic minority groups, our findings suggest that a focus on household food insecurity in urban settings is warranted. Public health nutrition programs should work with local social welfare programs to discuss resolutions to improve the affordability of core and healthy foods in general, and for fruits in particular to prevent unexpected health-related consequences for food insecure households.

## Abbreviations

AUD: Australian Dollar
BMI: Body Mass Index
CI: Confidence Interval
D8: District Eight
ELCSA: Latin American and Caribbean Household Food Security Scale
FAO: The Food Agriculture Organization
FSSM: Food Security Survey Module
HCMC: Ho Chi Minh City
IHPH: Institute of Hygiene and Public Health
OR: Odds Ratio
PVL: Poverty Line
VD: Vietnamese Dong

## References

[1] Basic definitions: Food insecurity. [http://www.fao.org/hunger/en/] [cited 3 April 2012]

[2] Ramsey R, Giskes K, Turrell G, Gallegos D (2011). Food insecurity among adults residing in disadvantaged urban areas: potential health and dietary consequences. Public Health Nutr.

[3] Holben DH, Pheley AM (2006). Obesity, diabetes, and blood pressure are greater in food insecure households in rural Appalachian Ohio. J Prev Chronic Dis.

[4] Eicher-Miller HA, Mason AC, Weaver CM, McCabe GP, Boushey CJ (2009). Food insecurity is associated with iron deficiency anemia in US adolescents. Am J Clin Nutr.

[5] Kirkpatrick SI, Tarasuk V (2008). Food insecurity is associated with nutrient inadequacies among Canadian adults and adolescents. J Nutr.

[6] Stuff JE, Casey PH, Szeto KL, Gossett JM, Robbins JM, Simpson PM (2004). Household food insecurity is associated with adult health status. J Nutr.

[7] Popkin BM (2009). The nutrition transition in low-income countries: an emerging crisis. Nutr Rev.

[8] Nhat TTH (2010). Tackling household food insecurity: the experience of Vietnam. Asian J Agric Dev.

[9] Mishra V, Ray R (2009). Dietary diversity, food security and undernourishment: the Vietnamese evidence. Asian Econ J.

[10] Suryanarayana MH, Silva D (2007). Is targeting the poor a penalty on the food insecure? Poverty and food insecurity in India. J Hum Dev.

[11] Hunger Statistics - Viet Nam. [http://www.fao.org/hunger/en/] [cited 3 April 2012]

[12] Food insecurity and vulnerability in Vietnam: Profiles of four vulnerable groups. [www.fao.org/es/esa] [cited 4 April 2012]

[13] Vietnam Government (2009). Resolution 63 on national food security.

[14] Cohen MJ, Garrett JL (2010). The food price crisis and urban food (in)security. Environ Urban.

[15] Ivanic M, Martin W (2008). Implications of higher global food prices for poverty in low-income countries. Agric Econ.

[16] Tran MH (2009). Food security and sustainable agriculture in Vietnam. Country Report: Vietnam.

[17] Thang NM, Popkin BM (2004). Patterns of food consumption in Vietnam: effects on socioeconomic groups during an era of economic growth. Eur J Clin Nutr.

[18] Melgar-Quinonez H, Hackett M (2008). Measuring household food security: the global experience. Rev Nutr Campinas.

[19] Guide to measuring household food security. [www.fns.usda.gov/fsec/files/fsguide.pdf] [cited 5 September 2011]

[20] Bermudez O, Palma De Fulladolsa P, Deman H, Melgar-Quinones H (2010). Food insecurity is prevalent in rural communities of El Salvador, Guatemala, Honduras and Nicaragua. FASEB J.

[21] Pérez-Escamilla R, Paras P, Hromi-Fiedler A (2008). Validity of the Latin American and Caribbean Household Food Security Scale (ELCSA) in Guanajuato, Mexico. J Fed Am Exp Biol.

[22] Pérez-Escamilla R (2009). The Latin American Household Food Security Measurement Scale (ELCSA). International Congress of Nutrition.

[23] Average population by districts. [http://www.pso.hochiminhcity.gov.vn/] [cited 15 October 2012].

[24] District 8 moves households living near channels. [www.sggp.org.vn/chinhtri/tientoidhd/2010/7/231776/] [10 October 2011]

[25] Donor bicycles for poor students in District 8. [http://www.congan.com.vn/?mod=detnews&catid=681&id=313036] [15 October 2011]

[26] Sullivan LM, Gartside M (2008). Power and sample size determination. Essentials of Biostatistics in public health.

[27] Ballantine NM. Purchasing determinants of food insecurity conditions amongst shoppers in Klipplaat. PhD thesis. Nelson Mandela Metropolitan University, Faculty of Arts; 2007.

[28] New poor and marginally poor household standard, period 2011–2015. [http://www.molisa.gov.vn/news/detail/tabid/75/newsid/52409/language/vi-VN/Default.aspx?seo=Chuan-ho-ngheo-can-ngheo-moi-giai-doan-2011-%E2%80%93-2015] [15 October 2011]

[29] Tang KH, Nguyen HHDT, Michael JD, David WS, Phan NTB, Tran TMH (2007). Overweight and obesity are rapidly emerging among adolescents in Ho Chi Minh City, Vietnam, 2002–2004. Int J Pediatr Obes.

[30] Acker T. Measuring food insecurity in Guatemala. PhD thesis. The Ohio State University, Department of Human Nutrition; 2011.

[31] Rivera JA, Barquera S, González-Cossío T, Olaiz G, Sepúlveda J (2004). Nutrition transition in Mexico and in other Latin American countries. Nutr Rev.

[32] Microfinance Poverty Assessment Tool. [http://www.cgap.org/gm/document-1.9.3004/TechnicalTool_05.pdf] [cited 11 June 2011]

[33] Cuong T, Dibley M, Bowe S, Hanh T, Loan T (2007). Obesity in adults: an emerging problem in urban areas of Ho Chi Minh City, Vietnam. Eur J Clin Nutr.

[34] Dang CV, Day RS, Selwyn B, Maldonado YM, Nguyen KC, Le TD (2010). Initiating BMI prevalence studies in Vietnamese children: changes in a transitional economy. Asia Pac J Clin Nutr.

[35] Kusama K, Le DSNT, Hanh TTM, Takahashi K, Hung NTK, Yoshiike N (2005). Reproducibility and validity of a food frequency questionnaire among Vietnamese in Ho Chi Minh City. J Am Coll Nutr.

[36] Cole TJ, Bellizzi MC, Flegal KM, Dietz WH (2000). Establishing a standard definition for child overweight and obesity worldwide: international survey. BMJ.

[37] Brittany GH, Ashley GM, Terry M, Tom H, Jodie LG (2011). Prevalence and predictors of food Insecurity in migrant farmworkers in Georgia. Am J Public Health.

[38] Gallegos D, Ellies P, Wright J (2008). Still there’s no food! Food insecurity in a refugee population in a refugee population in Perth, Western Australia. Nutr Diet.

[39] Nolan M, Rikard-Bell G, Mohsin M, William M (2006). Food insecurity in three socially disadvantaged localities in Sydney, Australia. Health Promot J Austr.

[40] Gopichandran V, Claudius P, Baby LS, Felinda A, Mohan VR (2010). Household food security in urban Tamil Nadu: a survey in Vellore. Natl Med J India.

[41] Melgar-Quinonez HR, Zubieta AC, MkNelly B, Nteziyaremye A, Gerardo MFD, Dunford C (2006). Household food insecurity and food expenditure in Bolivia, Burkina Faso, and the Philippines. J Nutr.

[42] Ahmed AU, Hill RV, Smith LC, Wiesmann DM, Frankenberger T, Gulati K (2007). The world’s most deprived: characteristics and causes of extreme poverty and hunger, 2020 vision for food.

[43] Vietnam consumers struggle with rising food prices. [http://news.monstersandcritics.com/business/news/article_1635177.php/Vietnam-consumers-struggle-with-rising-food-prices] [cited 11 June 2011]

[44] High food prices: Causes and possible actions. [www.ima.kth.se/utb/MJ1501/pdf/Engfeldt.pdf] [cited 11 July 2011]

[45] Vietnamese cultural profile. [http://ethnomed.org/culture/vietnamese/vietnamese-cultural-profile] [cited 1 February 2012]

[46] Scheier LM (2005). What is the hunger-obesity paradox?. J Am Diet Assoc.

[47] Tanumihardjo SA, Anderson C, Kaufer-Horwitz M, Bode L, Emenaker NJ, Haqq AM (2007). Poverty, obesity, and malnutrition: an international perspective recognizing the paradox. J Am Diet Assoc.

[48] Increasing fruit and vegetable consumption becomes a global priority. [http://www.fao.org/english/newsroom/focus/2003/fruitveg1.htm] [27 September 2012]

[49] Promoting fruit and vegetable consumption around the world. [http://www.who.int/dietphysicalactivity/fruit/en/] [cited 27 September 2012]

[50] Le CQ (2008). An empirical study of food demand in Vietnam. ASEAN Econ Bull.

[51] Popkin BM (1999). Urbanization, lifestyle changes and the nutrition transition. World Dev.

[52] Popkin BM (1998). The nutrition transition and its health implications in lower-income countries. Public Health Nutr.

[53] Business Monitor International. Vietnam Food & Drink Report Q4 2009. 2009:19–40.

[54] Agneta Y, Barrie M, Roger H, Marilyn T (2009). Food security - not just about rural communities in Africa and Asia. Public Health Nutr.

[55] Doak CM, Adair LS, Bentley M, Monteiro C, Popkin BM (2005). The dual burden household and the nutrition transition paradox. Int J Obes.

[56] Tran TMH, Komatsu T, Nguyen TKH, Nguyen VC, Yoshimura Y, Pham GT (2001). Nutritional status of middle-aged Vietnamese in Ho Chi Minh City. J Am Coll Nutr.

[57] Nyholm M, Gullberg B, Merlo J, Lundqvist-Persson C, Rastam L, Lindblad U (2007). The validity of obesity based on self-reported weight and height: Implications for population studies. Obesity.

[58] Isanaka S, Mora-Plazas M, Lopez-Arana S, Baylin A, Villamor E (2007). Food insecurity is highly prevalent and predicts underweight but not overweight in adults and school children from Bogota, Colombia. J Nutr.

[59] McIntyre L, Glanville NT, Officer S, Anderson B, Raine KD, Dayle JB (2002). Food insecurity of low-income lone mothers and their children in Atlantic Canada. Can J Public Health.

[60] Maynard LD, Galuska DA (2003). Maternal perceptions of weight status of children. Pediatrics.

[61] Scholtens SB, Brunekreef B (2007). Reported versus measured body weight and height of 4-year-old children and the prevalence of overweight. Eur J Pub Health.

[62] Wald E, Ewing L (2007). Parental perception of children’s weight in a paediatric primary care setting. Child Care Health Dev.

